# Serum Lipidomics Analysis of Classical Swine Fever Virus Infection in Piglets and Emerging Role of Free Fatty Acids in Virus Replication *in vitro*

**DOI:** 10.3389/fcimb.2019.00410

**Published:** 2019-12-03

**Authors:** Shengming Ma, Qian Mao, Wenxian Chen, Mengpo Zhao, Keke Wu, Dan Song, Xin Li, Erpeng Zhu, Shuangqi Fan, Lin Yi, Hongxing Ding, Mingqiu Zhao, Jinding Chen

**Affiliations:** College of Veterinary Medicine, South China Agricultural University, Guangzhou, China

**Keywords:** CSFV, lipidomics, free fatty acids, virus replication, IFN signaling

## Abstract

Lipids metabolism plays a significant role in cellular responses to virus pathogens. However, the impact of lipids metabolism in CSFV infection is not yet confirmed. In the present study, for the fist time, we performed serum lipidomics analysis of piglets infected with CSFV based on ultra-high performance liquid chromatography coupled with quadrupole time-of-flight mass spectrometry (UHPLC-QTOF-MS), and identified 167 differentially expressed lipid metabolites. Interestingly, free fatty acids (FFAs) accumulated significantly in these metabolites, accompanied by an increase in sphingolipids and a decrease in glycerolipids and glycerophospholipids, suggesting that CSFV infection markedly changed the serum lipid metabolism of piglets. FFAs are the principal constituents of many complex lipids and are essential substrates for energy metabolism. Based on this, we focused on whether FFAs play a prominent role in CSFV infection. We found that CSFV infection induced FFAs accumulation *in vivo* and *in vitro*, which is due to increased fatty acid biosynthesis. Meanwhile, we discovered that alteration of cellular FFAs accumulation by a mixture of FFAs or inhibitors of fatty acid biosynthesis affects progeny virus production *in vitro*. Furthermore, in the absence of glucose or glutamine, CSFV still has replication capacity, which is significantly reduced with the addition of fatty acid beta oxidation inhibitors, suggesting that the process of FFAs enter the mitochondria for beta oxidation to produce ATP is necessary for virus replication. Finally, we demonstrated CSFV induced FFAs accumulation results in impaired type I IFN signaling-mediated antiviral responses by down-regulating RIG-I-like receptors (RLRs) signaling molecules, which may represent a mechanism of CSFV replication. Taken together, these findings provide the first data on lipid metabolites during CSFV infection and reveal a new view that CSFV infection requires FFAs to enhance viral replication.

## Introduction

Classical swine fever virus (CSFV) is a member of the Pestivirus genus within the *Flaviviridae* family that is the causative agent of classical swine fever (CSF) in pigs and its genome consists of a single-stranded positive-sense genomic RNA of about 12.5 kb (Becher et al., 2003). CSF has a serious destructive effect on the immune and hematopoietic system, causing a series of clinical symptoms such as high fever, multiple hemorrhage, leukopenia, neurological dysfunction, abortion, and high mortality, which seriously endangers the healthy development of pig industry worldwide (Kleiboeker, 2002; Lohse et al., 2012). At present, CSF is largely controlled through mass vaccination because of limited treatment options (König et al., 1995; Moormann et al., 2000). Despite extensive efforts to control the spread of CSF disease through mass vaccination strategies, there is evidence that the emergence of CSFV with moderate or attenuated virulence leads to persistent recessive virulence and immunosuppression in pigs, which brings greater difficulties and challenges to the control and eradication of CSF (Edwards et al., 2000; Moennig, 2000; Stegeman et al., 2000). To develop new vaccines or specific drugs for effectively controlling infection, it is necessary to further understand the relationship between host and CSFV. Although numerous studies related to the mechanism of CSFV replication have been performed, the pathogenesis of CSFV is still poorly understood.

Lipids play an important role in regulating various life processes, not only as an important component of cells and internal organelle membranes, but also in regulating cell homeostasis in energy conversion, material transport, information recognition and signal transmission, cell development and differentiation, and cell apoptosis (Christie, 1978; Hadley, 1991). As an intracellular parasitic microorganism, viruses need lipid biogenesis participation in various steps of infection, such as viral replication, assembly, and energy supply (Bramhall and Wisnieski, 1981; Lorizate and Krausslich, 2011). Viruses hold specific classes of lipids and enrich them in the envelope structure to enhance their infectivity (Mercer and Helenius, 2008; Strating, 2012). In addition, viruses also alter lipid metabolism and provide favorable conditions for their replication (Nagy et al., 2016; Strating and Van Kuppeveld, 2017). More and more elementary studies show that abnormal lipid metabolism may be an important factor in the occurrence and development of many viral infectious diseases (Seo and Cresswell, 2013; Melanie, 2014). In recent years, the changes of lipid composition, lipid distribution, and lipid content in cell membranes and cells, and abnormal lipid metabolism have also attracted more and more attention and research as potential pathogenesis of various viral infectious diseases. Lipidomics is a comprehensive and systematic analysis and identification of lipids in cells and molecules interacting with them, which can be used as an effective tool for the discovery and subsequent identification of molecules associated with various diseases (Wenk and Markus, 2005; Sethi and Brietzke, 2017; Scott et al., 2018). It is helpful to explore the potential pathogenic mechanism of viruses by studying the lipidomics of viruses infected host cells. However, compared to genomics and proteomics, lipidomic studies of viruses, and their producer cells are limited.

Cellular lipids are abundant and diverse. Free fatty acids (FFAs) are the major constituents of many complex lipids and are essential substrates for energy metabolism (Yoshida et al., 1986; Hayyan et al., 2012). Fatty acids metabolism mainly includes *de novo* synthesis of fatty acids, oxidation of fatty acids, desaturation of fatty acids and elongation to produce fatty acids with different degrees of saturation and different carbon chain lengths (Watkins, 2013). Normally, cells mainly acquire fatty acids from dietary sources. However, in pathological cases, fatty acids in virus-infected or cancer cells are derived from fatty acid biosynthesis (Menendez and Lupu, 2007). A number of studies have reported that fatty acids have a prominent role on the replication of various viruses, including West Nile virus (WNV), rotaviruses (RV), hepatitis C virus (HCV), human immunodefciency virus type-1 (HIV-1), dengue virus (DENV), and respiratory syncytial virus (RSV) (Superti et al., 1995; Kapadia and Chisari, 2005; Hilmarsson et al., 2007; Heaton and Randall, 2010; Luchessi, 2010; Martin-Acebes et al., 2014). Chemicals inhibition of fatty acid synthesis by C75, TOFA, or triacsin C decreases the replication ability of these viruses (Yang et al., 2008; Miguel et al., 2011; Gaunt et al., 2013; Tang et al., 2014; Ohol et al., 2015; Kulkarni et al., 2017).

Our previous work has confirmed that CSFV rebuild cellular metabolic programs *in vitro*, thus aiding viral replication (Hongchao et al., 2017; Wenjie et al., 2017). However, systematic changes in lipid metabolites in CSFV-infected cells remain unknown. In the current study, and for the first time, we performed a serum lipidomics analysis of piglets infected with CSFV based on ultra-high performance liquid chromatography coupled with quadrupole time-of-flight mass spectrometry (UHPLC-QTOF-MS), as well as the differential lipids were identified. In particular, among these differential lipids, FFAs were significantly increased during CSFV infection. Moreover, we explored the effect of the FFAs on CSFV replication and type I IFN (interferon) signaling pathway by adding a mixture of FFAs (oleic: palmitoleic = 2:1) or disturbing the fatty acid biosynthase pathway with C75 and TOFA in CSFV-infected PK-15 and 3D4/2 cells. These studies provide the first data regarding the lipid metabolites during CSFV infection, which may represent potential anti-viral drug targets. Meanwhile, the current results demonstrated the important role of FFAs in CSFV replication, which may represent a mechanism of CSFV replication and CSFV induced immunomodulation.

## Results

### Establishment of a Platform for Serum Lipidomics Analysis in Piglets Infected With CSFV

**Figure 1:**
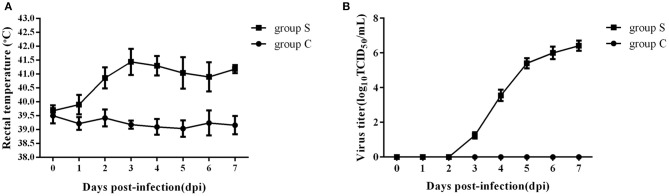
Changes of rectal temperature and blood viral load of piglets infected with CSFV. **(A)** Daily rectal temperature from 0 to 7 days after CSFV infection in piglets. **(B)** Daily blood viral load from 0 to 7 days after CSFV infection in piglets.

To investigate the effect of CSFV infection on lipid metabolism *in vivo*, experiments were carried out using piglets infected with Shimen, a representational virulent strain of CSFV. A total of ten 2-month-old piglets were randomly divided into two groups, one challenged with 10^5^ TCID_50_ of CSFV (Group S) and one inoculated with an equal volume of normal PK-15 cell-culture supernatant served as negative controls (Group C) (*n* = 5 each), and the rectal temperature and the blood viral load were daily detected after infection. As shown in Figure 1, group S piglets has a high fever from 39.4°C to 42.1°C (Figure 1A), and the blood viral load was first detected at 3 days post infection (dpi) and reached to a peak at 6 dpi (Figure 1B), while group C piglets had a stable rectal temperature and no virus was observed. During the whole infection period, the onset of clinical symptoms and autopsy lesions of group S piglets was consistent with the typical CSFV cases, while no significant changes in group C piglets. Thus, the typical disease form of CSF has been successfully established by shimen infection.

**Figure 2:**
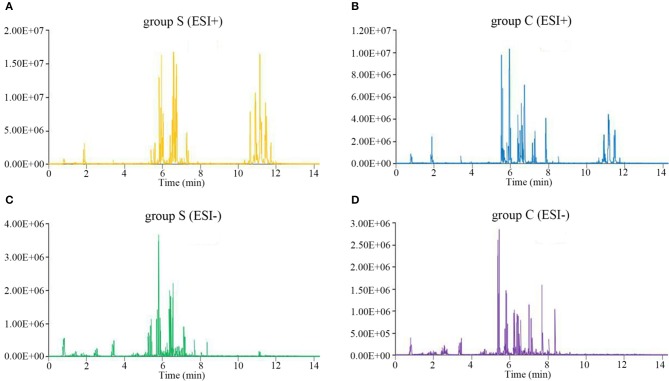
The typical base peak ion (BPI) chromatograms under the positive (ESI+) or negative ion mode (ESI-). **(A)** CSFV-infected piglets (ESI+). **(B)** DMEM-infected piglets (ESI+). **(C)** CSFV-infected piglets (ESI−). **(D)** DMEM-infected piglets (ESI−).

We next performed lipidomics analysis using UHPLC-QTOF-MS with lipids isolated from serum of group S and group C piglets at 6 dpi. The accurate m/z of precursors and product ions were matched against LipidBlast database and in-house standard library including retention time, accurate precursors, and product ions. The chromatographic retention of the same group samples will not change, and the signal of each substance is slightly different. As shown in Figure 2, we select a single representative from group S and group C samples and draw the typical base peak ion (BPI) chromatograms under the positive (ESI+) or negative ion mode (ESI-) to show the distribution of metabolite signals in the chromatogram. The threshold of matching similarity is >80% (Figure 2). These results suggest that the platform of UHPLC-QTOF-MS is reliable and can be utilized in the subsequent study.

### CSFV Infection Alters Serum Lipid Metabolism of Piglets

**Figure 3:**
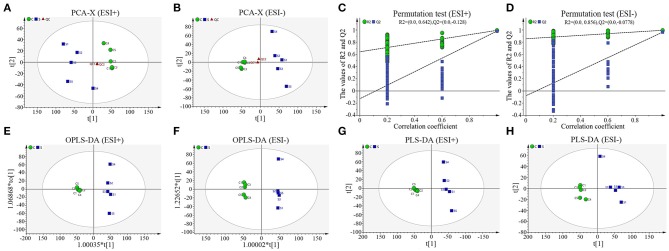
Sample score diagrams of group S and group C and OPLS-DA model replacement test diagrams under the positive (ESI+) or negative ion mode (ESI−). **(A)** The PCA score chart of group S and group C samples under ESI+. **(B)** The PCA score chart of group S and group C samples under ESI−. **(C)** The replacement test of group S and group C samples under ESI+. **(D)** The replacement test of group S and group C samples under ESI-. **(E)** The PLS-DA score chart of group S and group C samples under ESI+. **(F)** The PLS-DA score chart of group S and group C samples under ESI-. **(G)** The OPLS-DA score chart of group S and group C samples under ESI+. **(H)** The OPLS-DA chart plot of group S and group C samples under ESI-.

To truly reflect the lipidomics differences between group S and group C piglets, SIMCA (Version 14.1) software was performed to make the principal component analysis (PCA). Under the positive or negative ion mode, the PCA score chart showed that there was a significant trend of separation between group S and group C (Figures 3A,B). In order to eliminate the influence of background noise and thus highlight the differences between groups, the PLS-DA supervised multidimensional statistical analysis method was used to analyze the samples of group S and group C. In positive ion mode, a PLS-DA model with three effective principal components was established, R_2_X = 0.832, R_2_Y = 0.995, Q_2_ = 0.964 (Figure 3E). Similarly, in the negative ion mode, a PLS-DA model with two effective principal components was established, R_2_X = 0.74, R_2_Y = 0.994, Q_2_ = 0.79 (Figure 3F). The main parameters for judging the quality of the model are R_2_Y (model cumulative interpretation rate) and Q_2_ (model cumulative prediction rate). When R_2_Y is >0.5, it indicates that the current model is suitable for explaining the difference between the two groups; when the value of Q_2_ is >0.5, the current model is suitable for prediction. The replacement test showed that the current model is very reliable (Figures 3C,D). The PLS-DA score chart showed that there was significant spectral separation between group S and group C (Figures 3E,F). In order to further distinguish between the two groups of different substances, the OPLS-DA model was established in the positive or negative ion mode, respectively. In positive ion mode, the OPLS-DA model with one principal component and three orthogonal components is automatically established. The main quality parameters of the model are R_2_X = 0.885, R_2_Y = 0.999, Q_2_ = 0.963 (Figure 3G); in negative ion mode, one is automatically established. The main component and the OPLS-DA model of one orthogonal component, the main quality parameters of the model are R_2_X = 0.74, R_2_Y = 0.994, Q_2_ = 0.982 (Figure 3H). The OPLS-DA score chart indicated that the current model can distinguish very effectively between the two groups of samples in group C and group S (Figures 3G,H). All those data indicate that CSFV infection markedly changed the serum lipid metabolism of piglets.

### Identification of Serum Differential Lipids in Piglets Infected With CSFV

**Figure 4:**
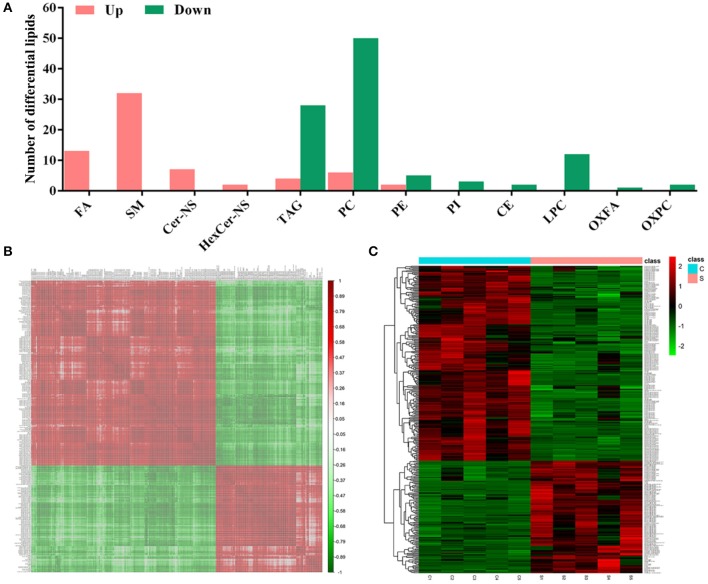
Differential metabolites between group S and group C. **(A)** Statistical analysis of differential metabolites between group S and group C. **(B)** Relevance matrix diagram of differential metabolites between group S and Group C. **(C)** Heat map of differential metabolites in Group S and Group C.

According to the criteria set for the variable importance in the projection (*VIP*) > 1 obtained from the PLS-DA model and *P*-value < 0.05 in the Student's *t*-test, totals of 167 differential lipids were identified between group S and group C. Among these differential lipids, 65 lipids were observed to be increased during CSFV infection, along with the simultaneous down-regulation of 102 lipids, respectively, in Tables S1, S2. The increased differential lipids mainly belonged to free fatty acids (FFAs), and sphingolipids including sphingomyelin (SM), ceramide non-hydroxyfatty acid-sphingosine (Cer-NS), and hexosylceramide non-hydroxyfatty acid-sphingosine (HexCer-NS). The decreased differential lipids mainly belonged to glycerolipids including cholesteryl ester (CE) and triacylglycerol (TAG), and glycerophospholipids including lysophophatidylcholine (LPC), phosphatidylcholine (PC), phosphatidylethanolamine (PE), phosphatidylinositol (PI), oxidized fatty acid (OXFA), and oxidized phosphatidylcholine (OXPC) (Figure 4A). Further, we performed a correlation matrix analysis of the differential compounds (*p* < 0.05) using R (version 3.4.1) software. To characterize the (concentration) correlation between the different metabolites, we performed a correlation (Pearson Correlation) analysis of the quantitative information for these substances, as showed in Figure 4B. Both rows and columns in the figure represent differential metabolites. The correlation coefficient metric is shown on the right side of the figure. The color shades of the squares in the figure are related to the correlation between the different metabolites. The differential lipids correlation matrix analyzed showed that there was no significant correlation between group S and group C (Figure 4B). In addition, we performed heat map analysis of differential compounds (*p* < 0.05) using R (version 3.4.1) software. The heat map analysis of the difference material is shown in Figure 4C. Each row represents a differential metabolite, each column represents a sample (number), the upper tree structure represents the similarity clustering relationship between samples, and the left tree structure represents the similarity between different metabolites class relationship. Heatmap analysis showed that all the differential lipids within group S or group C can be clustered together, but not between groups (Figure 4C). These results suggest that CSFV infection leads to abnormal expression of different lipids, which further indicate that CSFV infection causes lipid metabolism disorder.

### CSFV-Induced FFAs Accumulation Is Mainly Due to the Increase of Fatty Acid Synthesis

**Figure 5:**
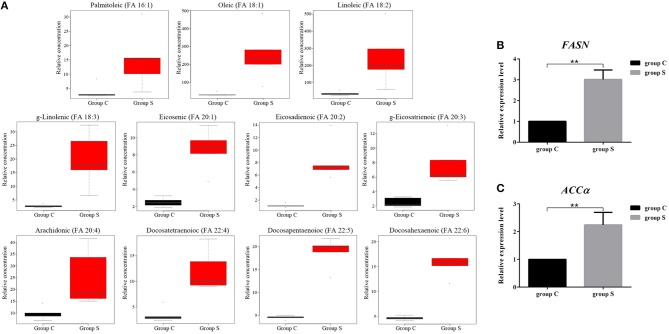
CSFV-induced FFAs accumulation is mainly due to the increase of fatty acid synthesis in serum of piglets. **(A)** Box diagram of the differentially expressed FFAs (*p* < 0.05) in Group S and Group C using R (version 3.4.1) software. **(B,C)** The transcription levels of FASN **(B)** and ACCα **(C)** in serum of Group S and Group C piglets were deteced by qRT-PCR as described in Materials and Methods. **P < 0.01. *P*-values were calculated using an One-way ANOVA test.

FFAs are the main components in the synthesis of different lipids and play an important role in the regulation of lipid metabolism (Yoshida et al., 1986; Hayyan et al., 2012). To characterize the difference FFAs between group S and group C, we used R (version 3.4.1) software to make box plot of differential compounds (*p* < 0.05). As shown in Figure 5A, all differential FFAs including palmitoleic (FA 16:1), oleic (FA 18:1), linoleic (FA 18:2), g-linolenic (FA 18:3), eicosenic (FA 20:1), eicosadienoic (FA 20:2), g-eicosatrienoic (FA 20:3), arachidonic (FA 20:4), docosatetraenoioc (FA 22:4), docosapentaenoioc (FA 22:5), and docosahexaenoic (FA 22:6) were significant increased during CSFV infection. In addition to the degradation of glycerides, phospholipids, sphingolipids, and steroids by lipase, another important source of intracellular FFAs is the increase in fatty acid synthesis. Previous studies have shown that FFAs in virus-infected cells or cancer cells are mainly derived from fatty acid biosynthesis (Menendez and Lupu, 2007). To determine whether CSFV-induced FFAs accumulation is due to the increase of fatty acid biosynthesis, we used quantitative real time PCR (qRT-PCR) to evaluate the mRNA expression levels of the key enzymes in fatty acid biosynthesis in serum of group S and group C piglets at 6 dpi, including fatty acid synthase (FASN) and acetyl CoA carboxylase alpha (ACCα). Results showed that the transcription levels of FASN and ACCα were significantly increased in group S piglets as compared to group C piglets (Figures 5B,C), indicating that CSFV infection increased fatty acid biosynthesis, which may leads to the accumulation of FFAs.

### CSFV-Induced FFAs Accumulation and Increased Fatty Acid Synthesis *in vitro*

**Figure 6:**
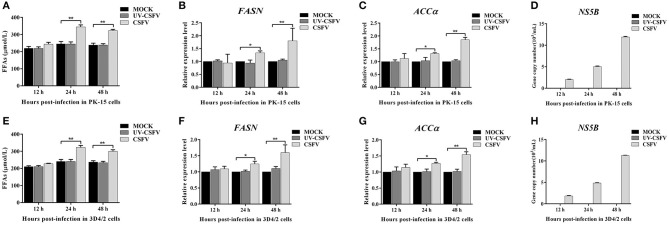
CSFV-induced FFAs accumulation and increased fatty acid synthesis *in vitro*. PK-15 and 3D4/2 cells were mock-infected or infected with CSFV (MOI = 1.0) or UV-inactivated CSFV (MOI = 1.0) for 12, 24, and 48 h. **(A,E)** the levels of FFAs in the supernatant of PK-15 **(A)** and 3D4/2 **(E)** cells were detected by FFAs ELISA Kit. **(B,C,F,G)** The transcription levels of FASN, ACCα in PK-15 and 3D4/2 cells were detected by qRT-PCR as described in Materials and Methods. **(D,H)** CSFV protein NS5B was detected by qRT-PCR to evaluate the progression of viral infection in PK-15 **(D)** and 3D4/2 **(H)** cells. The data represent the mean ± SD of 3 independent experiments. *P < 0.05; **P < 0.01. *P*-values were calculated using an One-way ANOVA test.

To further investigate the roles of cellular FFAs in CSFV infection, we chose PK-15 and 3D4/2 cell lines as cell models of CSFV infection *in vitro*, and examined the levels of FFAs in cell supernatants at different hours post infection (hpi) with CSFV using FFAs ELISA Kit (Zcibio, ZC-39969). As shown in Figures 6A,E, the levels of FFAs in the supernatant of cells infected with CSFV increased significantly at 24 and 48 hpi. Further, we detected the expression levels of FASN and ACCα in cells using qRT-PCR. Results showed that the mRNA levels of FASN and ACCα observed an increased tendency from 24 to 48 hpi (Figures 6B,C,E,F), suggesting that cellular up-regulated fatty acid biosynthesis was responsible for FFAs accumulation following CSFV infection. More importantly, we used ultraviolet (UV)-inactivated CSFV to evaluate whether FFAs can be up-regulated following treatment. Results showed that no significant changes in the relative levels of FFAs, FASN and ACCα were observed during UV-CSFV infection (Figures 6A–C,E–G), indicating that FFAs is required for effective viral replication. Meanwhile, CSFV protein NS5B was detected by qRT-PCR to evaluate the progression of viral infection, result shown that the transcription level of NS5B gene was detected only in CSFV-infected PK-15 and 3D4/2 cells, and increased gradually with the prolongation of infection time, while the expression of NS5B gene was not detected in mock and UV-infected cells (Figures 6D,H).

### FFAs Are Required for CSFV Replication *in vitro*

**Figure 7:**
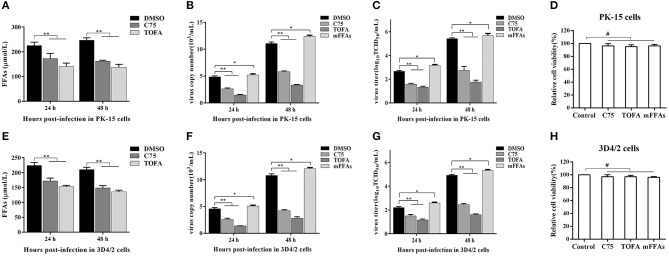
FFAs are required for CSFV replication *in vitro*. **(A,E)** CSFV-infected PK-15 and CSFV-infected 3D4/2 cells were treated with C75 (20 μM), TOFA (30 μM) or DMSO for 24 and 48 h. The levels of FFAs in the supernatant of PK-15 **(A)** and 3D4/2 **(E)** cells were detected by FFAs ELISA Kit. **(B,C,F,G)** CSFV-infected PK-15 and CSFV-infected 3D4/2 cells were treated with mFFAs, C75 (20 μM), TOFA (30 μM), or DMSO for 24 and 48 h. The virus copy number of CSFV in PK-15 **(B)** and 3D4/2 **(F)** cells were detected by qRT-PCR. The titer of CSFV in PK-15 **(C)** and 3D4/2 **(G)** cells were detected by IFA. **(D,H)** PK-15 and 3D4/2 cells were treated with mFFAs (100 μM), C75 (20 μM), TOFA (30 μM), or DMSO for 48 h. PK-15 **(D)** and 3D4/2 **(H)** cells viability were detected by CCK-8 Cell Counting Kit. The data represent the mean ± SD of 3 independent experiments. *P < 0.05; **P < 0.01; ^#^*P* > 0.05. *P*-values were calculated using an One-way ANOVA test.

To further determine the effect of FFAs on CSFV replication, fatty acid biosynthesis inhibitor C75 (20 μM) and TOFA (30 μM) were used to treat CSFV-infected PK-15 or 3D4/2 cells, respectively, and DMSO treatment was used as a negative control. After treatment for 24 and 48 h (h), we examined the levels of FFAs in cell supernatants and analyzed the capability of CSFV replication by detecting the virus copy number and titer. Compared with DMSO treatment, both C75 and TOFA significantly inhibited the accumulation of FFAs (Figures 7A,E) and reduced CSFV copy number and titer (Figures 7B,C,F,G), showing that fatty acid biosynthesis inhibition reduce FFAs accumulation and CSFV production. However, when we treated cells with a mixture of FFAs (mFFAs) at a 2:1 ratio of oleic to palmitoleic (100 μM), the capability of CSFV replication increased (Figures 7B,C,F,G). Meanwhie, in order to exclude the effect of fatty acid biosynthesis inhibitors and mFFAs on viral replication by altering cell viability, CCK-8 Cell Counting Kit (Vazyme,A311-01) was used to evaluate the effects of C75, TOFA and mFFAs on PK-15 and 3D4/2 cells viability. Statistical analyses revealed no significant effects on the viability of cells treated with C75, TOFA or mFFAs (*P* >0.05) (Figures 7D,H). Taken together, these data revealed that FFAs are required for efficient CSFV replication.

### FFAs Are an Indispensable Source of ATP for CSFV Replication

**Figure 8:**
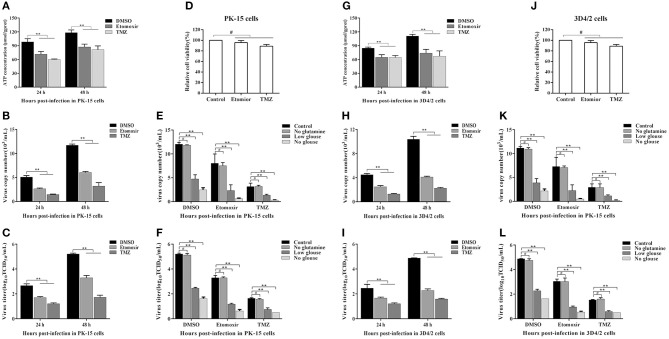
Free fatty acid is an indispensable source of ATP for CSFV replication *in vivo*. **(A–D,G–J)** CSFV-infected PK-15 and CSFV-infected 3D4/2 cells were treated with etomoxir (2 μM), TMZ (60 μM), or DMSO for 24 and 48 h. The levels of ATP in PK-15 **(A)** and 3D4/2 **(G)** cells were detected by ATP assays Kit. The virus copy number of CSFV in PK-15 **(B)** and 3D4/2 **(H)** cells were detected by qRT-PCR. The titer of CSFV in PK-15 **(C)** and 3D4/2 **(I)** cells were detected by IFA. PK-15 **(D)** and 3D4/2 **(J)** cells viability were detected by CCK-8 Cell Counting Kit. **(E,F,K,L)** CSFV-infected PK-15 and 3D4/2 cells were cultured for 48 h in glucose-free media or glucose-low media or glutamine-free media or complete media, respectively, along with the treatment of etomoxir (2 μM) or TMZ (60 μM) or DMSO. The virus copy number of CSFV in PK-15 **(E)** and 3D4/2 **(F)** cells were detected by qRT-PCR. The titer of CSFV in PK-15 **(K)** and 3D4/2 **(L)** cells were detected by IFA. The data represent the mean ± SD of 3 independent experiments. **P < 0.01; ^#^*P* > 0.05. *P*-values were calculated using an One-way ANOVA test.

FFAs not only participate in the formation of cell structure, but also provide an energy source for host cells through fatty acid beta oxidation (FAO) (Yoshida et al., 1986; Hayyan et al., 2012). Etomoxir is an inhibitor of carnitine palmitoyltransferase A (CPT1), which is required for the oxidation of long-chain acyl CoA esters. Trimetazidine (TMZ) reduces acetyl coenzyme A produced by free fatty acid metabolism, thereby stimulating pyruvate dehydrogenase and indirectly enhancing glucose oxidation. To directly analyze whether the increased FFAs during CSFV infection provides ATP for virus replication through FAO, we tested the levels of ATP in PK-15 and 3D4/2 cells and the replication ability of CSFV following etomoxir (2 μM) or TMZ (60 μM) treatment. We found that the pharmacological alteration of FAO with etomoxir and TMZ not only reduced the levels of cellular ATP (Figures 8A,G), but also significantly decreased the capability of CSFV replication (Figures 8B,C,H,I). Meanwhile, there are no significant effects on the viability of PK-15 or 3D4/2 cells treated with etomoxir or TMZ (Figures 8D,J). These findings suggest that CSFV-induced the accumulation of FFAs was transported to mitochondria for beta oxidation, thus providing ATP for viral replication.

As another energy source, glucose is the most widely used carbon source for energy metabolism and biosynthesis in mammalian cells. Studies have found that most viral infections cause glycolysis to provide energy for viral replication (Fontaine et al., 2014; Findlay and Ulaeto, 2015). To further determine whether FAO contribute to productive CSFV infection, CSFV-infected PK-15 and 3D4/2 cells were cultured for 48 h in glucose-free media or glucose-low media or glucose-high media, respectively, along with the treatment of etomoxir or TMZ or DMSO. As show in Figures 8E,F,K,L, the capability of CSFV replication is dependent on a certain glucose concentration, and the higher glucose concentration, the stronger capability of CSFV replication (Figures 8E,F,K,L). Interestingly, the virus still has a strong replication capacity under conditions of low glucose or glucose loss, which is significantly reduced when treated with FAO inhibitors (Figures 8E,F,K,L). In addition, glutamine can replace glucose in the TCA cycle to provide energy for viral replication (Sanchez et al., 2017). However, in the absence of glutamine, CSFV replication had no significant changes relative to the complete medium (Figures 8E,F,K,L). Together, these results suggested that ATP produced by FFAs transported to mitochondria for FAO is essential for CSFV replication.

### CSFV Induced FFAs Accumulation Results in Impaired Type I IFN Signaling-Mediated Antiviral Responses

**Figure 9:**
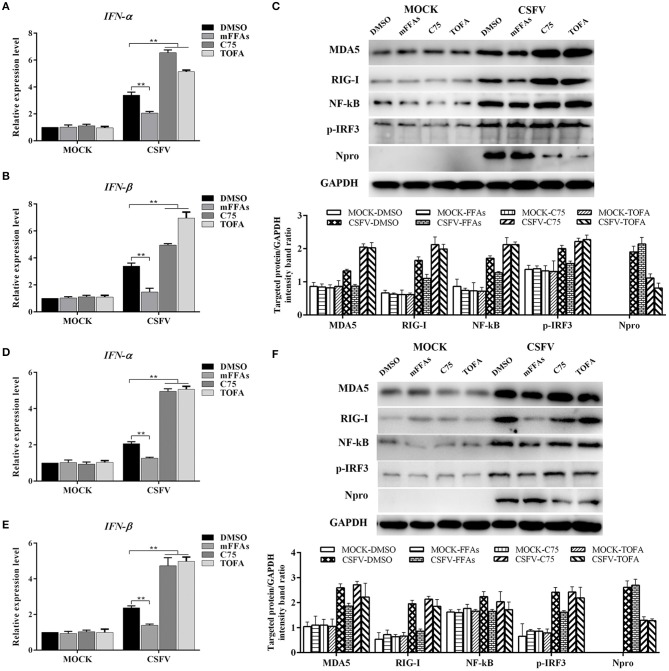
CSFV induced FFAs accumulation suppressed type I IFN expression by down-regulating RLR signaling pathway. CSFV-infected or mock-infected PK-15 and 3D4/2 cells were treated with mFFAs (100 μM), C75 (20 μM), TOFA (30 μM) or DMSO for 48 h. **(A,B,D,E)** The transcription levels of IFNα and IFN-β in PK-15 and 3D4/2 cells were detected by qRT-PCR as described in Materials and Methods. **(C,F)** The protein expression levels of MDA5, RIG-I, NF-kB, p-IRF3, Npro and GAPDH (control) in PK-15 **(C)** and 3D4/2 **(F)** cells were examined by western blot. The relative levels of the targeted proteins were estimated by densitometric scanning, and the ratios were calculated relative to the GAPDH control. The data represent the mean ± SD of 3 independent experiments. **P < 0.01. *P*-values were calculated using an One-way ANOVA test.

According to the current understanding of the pathogenesis of CSFV, we know that CSFV infection inhibits the production of type I IFN *in vitro*, thereby resulting in the persistent survival of CSFV in host cells (Bensaude, 2004). Studies have shown that the accumulation of FFAs in HCV-infected cells inhibits the activation of type I IFN signaling pathway, resulting in impaired antiviral response (Gunduz et al., 2012). To further understand the mechanism of FFAs affecting the replication of CSFV, mFFAs and fatty acid biosynthesis inhibitors were used to regulate the production of FFAs in CSFV-infected or mock-infected PK-15 and 3D4/2 cells, and the mRNA expression levels of IFN-α and IFN-β were detected by qRT-PCR to verify if alteration of cellular FFAs production has an effect on the IFN signaling pathway. Results showed a significant decrease in mRNA expression of IFN-α and IFN-β genes in mFFAs treated CSFV-infected PK-15 and 3D4/2 cells for 24 and 48 hpi, and a increase in C75 or TOFA treated CSFV-infected cells, compared with DMSO treated CSFV-infected cells (Figures 9A,B,D,E), suggesting that CSFV-induced FFAs accumulation may affect viral replication by suppressing type I IFN production. CSFV can be recognized by the members of the RIG-I-like receptors (RLRs) in cells, such as melanoma differentiation-associated gene 5 (MDA5) and retinoic acid-inducible gene I (RIG-I), which mediate type I IFN production by NF-κB and phosphorylation of IRF3 (Dong et al., 2013). Based on this, we suspect that FFAs may suppress type I IFN production by affecting the activation of RLRs signaling pathway. To test this conjecture, key markers of RLRs signaling pathway, including RIG-I, MDA5 NF-κB and p-IRF3 (phosphorylated IRF3), were examined by western blot in CSFV-infected or MOCK-infected PK-15 or 3D4/2 cells treated with mFFAs, C75, TOFA, or DMSO for 48 hpi. Surprisingly, our results showed that the expression of RIG-I and MDA5 proteins in CSFV-infected PK-15 and 3D4/2 cells were significantly increased in C75 or TOFA treated CSFV-infected cells, and NF-κB and p-IRF3 was activated, contrary to the results of mFFAs treated CSFV-infected cells, compared with DMSO treated CSFV-infected cells (Figures 9C,F). However, there is no significant difference in expression of these proteins in mock-infected cells treated with mFFAs, C75 or TOFA, compared with DMSO treated mock-infected cells (Figures 9C,F). Meanwhile, CSFV non-structural protein Npro, which is associated with virus replication, was detected by western blot to estimate the progression of infection, we found that Npro protein displayed a higher level in FFAs treated CSFV-infeted cells than in DMSO treated CSFV-infeted cells, whereas Npro protein in C75 or TOFA treated CSFV-infected cells showed a lower Level (Figures 9C,F). These findings indicate that CSFV-induced FFAs accumulation suppressed type I IFN production by down-regulating RLR signaling, thereby resulting in the persistent survival of CSFV in host cells.

## Discussion

It is well-known that viruses alter host metabolism, especially lipid metabolism, to facilitate their infection and replication (Heaton and Randall, 2011). Like other members of the *Flaviviridae* family, CSFV can also cause cellular metabolic disorders and lead to a series of pathological reactions, such as high fever, multiple hemorrhage, leukopenia and inflammation (Kleiboeker, 2002; Lohse et al., 2012; Hongchao et al., 2017). In the current study, we provided for the first time observations on the lipids changes in the serum of CSFV-infected piglets based on UHPLC-QTOF-MS, which might provide suitable targets for drug intervention and therapeutic vaccines. More importantly, we found that FFAs accumulation plays a positive role in CSFV infection *in vitro*, which may reveal a new view for the pathogenesis and immune escape mechanism of CSFV.

Interestingly, viruses from same family induce some similar lipids changes. During CSFV infection, the increased lipids were mainly classified as FFAs and sphingolipids, while the reduced lipids were mainly classified as glycerolipids and glycerophospholipids (Figures 2–5) (Tables S1, S2). The accumulation of FFAs caused by viral infection contributes to the formation of membrane viral replication complexes and promotes the transmission of viruses, which have been confirmed in DENV and HCV (Kapadia and Chisari, 2005; Heaton and Randall, 2010). Sphingolipids, including sphingomyelin and ceramide, play an important role in the adsorption, assembly and intracellular transport of HCV and HBV, and are essential for HCV and HBV replication (Merrill et al., 2001). Glycerolipids, are an important component of cell membrane and participates in virus infection and release. Removal of cell membrane cholesterol can promote the release of virus from infected cells, but the infectivity of released virus particles is reduced (Popescu and Dubuisson, 2010). Gglycerophospholipids is considered to be an important regulator of inflammation and cell proliferation (Popescu and Dubuisson, 2010). However, whether these changed lipids play the same role in the infection of CSFV and other *Flaviviridae* family viruses still needs further confirmation.

FFAs are the major constituents of many complex lipids and are essential substrates for energy metabolism. To better understand the roles of cellular FFAs in CSFV infection, we analyzed the physiological significance of FFAs in PK-15 and 3D4/2 cells *in vitro*. The PK-15 cell line is usually used as the model cell for studying CSFV infection (Grummer et al., 2006; Sun et al., 2008), while 3D4/2 cell line is representative of the macrophage that is the target for CSFV infection (Knoetig et al., 1999). When the target cells were infected with CSFV, we found that CSFV infection promoted FAAs accumulation (Figures 6A,E). This observation was consistent with a previous report that CSFV infection promotes the substantial accumulation of long chain saturated and unsaturated fatty acids (Wenjie et al., 2017). In pathological cases, FFAs in some envelope virus-infeted cells are derived from fatty acid biosynthesis (Yang et al., 2008; Miguel et al., 2011; Gaunt et al., 2013; Tang et al., 2014; Ohol et al., 2015). From gene expression analysis, FASN and ACCα expression levels, as the key enzymes regulating fatty acid synthesis, were found markedly increased in CSFV-infected cells (Figures 6B,C,F,G), which was consistent with the reports of HCV, HCMV, and DENV (Kapadia and Chisari, 2005; Heaton and Randall, 2010; Spencer et al., 2011), but contrary to WNV and KSHV (Delgado et al., 2012; Martin-Acebes et al., 2014).

Recent advances indicated that several members of the *Flaviviridae* family utilize host-cell machinery to create a specific optimal lipid microenvironment for assembly of their replication complex, where FFAs seems to play an essential role (Heaton and Randall, 2011). To further determine whether the accumulation of FFAs play a prominent role in the replication of CSFV, we explored the effect of the FFAs on CSFV replication by adding a mixture of FFAs (oleic: palmitoleic = 2:1) or disturbing the fatty acid biosynthase pathway with C75 and TOFA in CSFV-infected PK-15 and 3D4/2 cells. As showed in Figure 7, we revealed that the accumulation of FFAs and the activation of fatty acid biosynthesis are required for effective CSFV replication (Figure 7). It should be noted that fatty acid biosynthesis inhibitors of C75 and TOFA may provide novel drug targets for the treatment of CSFV as they inhabit CSFV replication. Available evidence suggests that FFAs can be transferred to mitochondria for beta-oxidation to produce acetyl CoA, which is a key component of the TCA cycle, thus driving oxidative phosphorylation and ATP production (Yoshida et al., 1986; Hayyan et al., 2012). In addition, glucose and glutamine, as the main carbon and nitrogen sources, can also provide the necessary energy for virus replication. Recent studies showed that glycolysis, glutaminolysis, and FAO are all required for maximal KSHV virus production (Sanchez et al., 2017). However, the presence of glucose is not important for vaccinia virus replication unless beta-oxidation of fatty acids is inhibited, whereas glutamine is essential (Greseth and Traktman, 2014). In our study, we showed that the process of FFAs enter the mitochondria for beta oxidation to produce ATP is necessary for CSFV replication (Figure 8). Meanwhile, We also found that glucose appears to be very important for CSFV replication, whereas glutamine is dispensable (Figure 8). These studies suggest that significant differences between the energy sources for viral replication exist between different viruses, which may be related to host specificity and different pathogenic mechanisms of infections.

It is worth mentioning that FFAs production is part of many signal transduction pathways in the cell, including regulating the IFN response (Schoggins and Randall, 2013). Previous studies have shown that CSFV infection can lead to impaired interferon response, which is beneficial to the sustained survival of the virus in host cells (Bensaude, 2004). To this end, we asked whether CSFV-induced FFAs accumulation modulates type I IFN production, this was important to reveal the molecular mechanism of FFAs affecting the replication of CSFV. As showed in Figure 9, we have documented an decrease in the mRNA level of IFN-α and IFN-β in mFFAs treated CSFV-infected PK-15 and 3D4/2 cells, but a significant increase in C75 or TOFA treated CSFV-infected cells, compared with DMSO treated CSFV-infected cells (Figures 9A,B,D,E). This result was consistent with a previous study, in which the accumulation of FFAs in HCV-infected cells inhibits the activation of type I IFN signaling pathway, resulting in impaired antiviral response (Gunduz et al., 2012). Importantly, CSFV can be recognized by the members of the RLRs in cells, such as RIG-I and MDA5, which mediate type I IFN production by phosphorylation of IRF3 or activation of NF-κB (Dong et al., 2013). Further, key markers of RLRs signaling pathway, including RIG-I, MDA, NF-κB and p-IRF3, were examined by western blot. We demonstrated that the expression of RIG-I and MDA5 proteins in CSFV-infected PK-15 and 3D4/2 cells were significantly decreased in C75 or TOFA treated cells, and NF-κB and p-IRF3 were activated, contrary to the results of mFFA treated CSFV-infected cells, compared with DMSO treated CSFV-infected cells (Figures 9C,F). Together, these findings suggest that CSFV-induced FFAs accumulation suppressed type I IFN production by inhibiting RLR signaling, thereby resulting in the persistent survival of CSFV in host cells.

In conclusion, our study demonstrated CSFV infection causes lipid metabolism disorders in host cells and provided the first data regarding the lipid metabolites during CSFV infection, which may provide a scientific basis for the study of potential antiviral drug targets. At the same time, we proved that CSFV-induced FFAs accumulation is beneficial to virus infection, which can provide the necessary ATP for virus replication and suppressed type I IFN production by inhibiting RLR signaling, thereby resulting in the persistent survival of CSFV in host cells. All these findings contribute to our understanding of the critical role of lipids in CSFV infection, but are still insufficient. It is still necessary to explore the molecular mechanisms of the interplay between CSFV and lipids, so as to understand the pathogenic mechanism of CSFV more comprehensively, and provide a solid scientific basis for the treatment and prevention of CSFV.

## Materials and Methods

### Reagents and Antibodies

4-Methylene-2-octyl-5-oxotetrahydrofuran-3-carboxylic acid (C75) (sc-202511), 5-Tetradecyloxy-2-furonic acid (TOFA) (sc-200653), and etomoxir (sc-208284) were purchased from Santa Cruz Biotechnology. Trimetazidine (TMZ) (S61054) was purchased from Shyuanye Biotechnology. High glucose dulbecco's modified eagle medium (DMEM) (11995040), low glucose DMEM (11885084), no glucose DMEM (11885084), high glucose DMEM with no glutamine (11960069), RPMI 1640 medium (21875091), glucose free RPMI 1640 medium (11879020), and glutamine free RPMI 1640 medium (21870076) were obtained from Gibco. The following primary antibodies were used in the study: rabbit monoclonal anti-MDA5 (Sigma-Aldrich, SAB2101127), rabbit polyclonal anti-RIG-I (Cell Signaling Technology, 3743), rabbit polyclonal anti-NF-κB (Beyotime, AF0246), rabbit monoclonal anti-Phospho-IRF3 (Beyotime, AF1594), and mouse monoclonal anti-GAPDH (Beyotime, AG019) and mouse monoclonal anti-CSFV E2 (WH303) (JBT, 9011). Mouse polyclonal anti-CSFV Npro was kindly provided by Dr. Xinglong Yu (Veterinary Department, Hunan Agricultural University, China). Te secondary antibodies were used in the study: HRP-conjugated goat anti-mouse IgG (Beyotime, A0192), HRP-conjugated goat anti-rabbit IgG (Beyotime, A0208) and Alexa Fluor 488-labeled Goat Anti-Mouse IgG (Beyotime, A0428).

### Cell Culture and Virus Infection

The swine kidney cell line PK-15 (ATCC, CCL-33) and porcine macrophage cell line 3D4/2 (ATCC, CRL-2845) were cultured in this study. PK-15 cells were maintained in DMEM containing with 10% fetal bovine serum (FBS, Gibco) 1% penicillin-streptomycin solution. 3D4/2 cells were grown in RPMI 1640 media supplemented with 10% FBS and 1% penicillin-streptomycin solution. The cells were cultured at 37°C with 5% CO_2_. The virulent CSFV strain Shimen was used in the present study and tittered as described previously (Hongchao et al., 2017). All cells with 80% confluences in cell culture plates were infected in serum-free DMEM medium with CSFV at a multiplicity of infection (MOI) of 1 for 2 h, after which the medium was replaced with complete DMEM or RPMI 1640 containing 2% FBS. The cells were then cultured at 37°C with 5% CO_2_ for a different hour post infection (hpi).

### Animal Experiments

All procedures were conducted following regulations of the Laboratory Animal Ethics Committee of South China Agricultural University. Briefly, a total of ten 2-month-old piglets, without PRRSV, PRV, PPV infection, were randomly divided into two groups, one challenged with 10^5^ TCID_50_ of CSFV (Group S) and one inoculated with an equal volume of normal PK-15 cell-culture supernatant served as negative controls (Group C) (*n* = 5 each). Two groups were separately maintained in isolators with filtered air of positive pressure in a SPF animal facility. Rectal temperatures were recorded each morning and animals were observed daily for clinical signs. After CSFV infection, the anterior vena cava blood of piglets was sterilely collected into heparin sodium anticoagulant tubes every other day. Then, the blood samples were centrifuged immediately and serum was harvested and stored at −80°C for further study.

### Serum Lipidomics Analysis With UHPLC-QTOF-MS

All samples were analyzed based on UHPLC-QTOF-MS non-targeted lipidomics platform. The project includes sample preparation, UHPLC-QTOF-MS analysis, raw data preprocessing, univariate and multivariate statistical analysis, and identification of differential lipids. UHPLC-QTOF-MS analysis was carried out in positive and negative ion mode, i.e., positive and negative electrospray ionization (ESI) mode. ESI is a fixed ionization mode. Positive (ESI+) and negative ions (ESI-) are our scanning mode for ions. In the process of ionization, both positive and negative ions are produced simultaneously. The selection of scanning mode (as in the range of m/z, can play a screening role). If ESI+ mode is selected, negative ions will be filtered out and can not be collected by the monitor. On the contrary, ESI- mode is adopted. Positive ions are filtered out, and the combination of the two modes will widen the range of metabolites detected and screened.

A total of 50 μL serum sample was mixed with 230 μL of ice-cold methanol/water (8:15, v/v) and 400 μL MTBE (methyl tert-butyl ether). The mixtures were vortexed for 1 min and standing for 2 h at 4°C. Then it was centrifugated at 3,000 rpm for 15 min. A 260 μL of supernatant was dried under gentle nitrogen stream, and re-dissolved in 100 μL of dichloromethane/methanol (1:1, v/v) before UHPLC-QTOF-MS analysis. The injection volume is 2 μL(ESI+)/6 μL(ESI-). Quality control (QC) sample pooled from the extractions of all samples were prepared and analyzed with the same procedure as that for the experiment samples.

Chromatographic separation was performed on an Agilent UHPLC system (1290) with a Phenomenex Kinetex C18 column (2.1 × 100 mm, 1.7 μm) at a flow rate of 0.3 mL/min and 40°C column temperature. The mobile phases consisted of water (phase A) and acetonitrile (phase B), both with 0.1% formic acid (v/v). A linear gradient elution was performed with the following program: 0 min, 40% B; 12 min, 100% B; 13.5 min, 100% B; 13.7 min, 40% B and held to 18 min.

The eluents were analyzed in positive ion mode on a hybrid quadrupole time-of-light mass spectrometer (Triple TOF 5600 system, AB Sciex, Comcord, ON, Canada) equipped with a DuoSpray ion source. A typical information dependent acquisition comprising the acquisition of a survey TOF MS spectrum and then a MS/MS experiment was applied in the analysis. The TOF MS scan was operated under the high-resolution settings with a range of 60–1,000 m/z and an accumulation time of 200 ms. The software for controlling instrument and collecting data was Analyst TF 1.7 (AB Sciex, Comcord, ON, Canada).

The raw data of UPLC-QTOF-MS were firstly transformed to mzXML format by ProteoWizard and then processed by XCMS and CAMERA packages in R software platform. The final data was exported as a peak table file, including observations (sample name), variables (rt_mz), and peak areas. The data was normalized against total peak areas before performing univariate and multivariate statistics.

For multivariate statistical analysis, the normalized data were imported to SIMCA software (version 13.0, Umetrics, Umeå, Sweden), where the data were preprocessed by Pareto scaling and mean centering before performing PCA, PLS-DA, and OPLS-DA. For univariate statistical analysis, the normalized data were analyzed by Welch's *t* test on the variables of normal distribution, or by Wilcoxon Mann-Whitney test on the variables of abnormal distribution. The variables with VIP values of PLS-DA or OPLS-DA model larger than 1 and *p* values of univariate statistical analysis lower than 0.05 were identified as potential differential metabolites.

### Quantitative Real-Time PCR

The expression levels of FASN, ACCα, and NS5B were determined by qRT-PCR. According to the manufacturer's protocol of TRIzol RNA extraction kit (Life Technologies, USA), total RNA from serum samples of group S and group C piglets and CSFV-infected PK-15 and 3D4/2 cells cultured in different environments were extracted. And synthesis of cDNA was performed using the PrimeScriptTM RT reagent Kit with gDNA Eraser (TAKARA, DRR036). The primer pairs specific for pigs FASN, ACCα, IFNα, IFN-β, β-actin, and CSFV NS5B genes (Table 1) were used for the qPCR amplifications utilizing iQ5 iCycler detection system (Bio-Rad) following the instructions of SYBR® Premix Ex TaqTM II (Tli RNaseH Plus) (TAKARA, DRR082). Relative quantification of the FASN, ACCα, IFNα and IFN-β expression were calculated using 2^−ΔΔct^ method. The viral copy number was calculated using absolute quantification method by constructing a standard curve of recombinant plasmid containing CSFV NS5B Gene. Three biological replicates were used for each cell sample, and three qPCR reactions were performed for each replicate sample and the average value was obtained.

**Table 1:** Oligonucleotide primer sequences for real-time qRT-PCR.

**No**.	**Target gene**	**Genbank Acc. No**.	**Primer name**	**Type**	**Sequence(5^**′**^-3^**′**^)**	**Amplicon (bp)**
1	FASN	NM_001099930.1	qRT-PCR-FASN-F	5′	CCAGCATCACCATAGACACGG	146
			qRT-PCR-FASN-R	3′	CATGAATTGCAGCGAGGAGTTAG	
2	ACCα	NM_001114269.1	qRT-PCR-ACCα-F	5′	GAATACCCGTGGGAGTAGTTGC	266
			qRT-PCR-ACCα-R	3′	CACGATGTAAGCGCCGAAC	
3	NS5B	EF026757.1	qRT-PCR-NS5B-F	5′	ACTCATCAGGATCCCCCTCAC	254
			qRT-PCR-NS5B-R	3′	CTTACTTGTATTGGTGTATGGGAGC	
4	IFN-α	NM_214393.1	qRT-PCR-IFN-α-F	5′	CTCAGCCAGGACAGAAGCA	108
			qRT-PCR-IFN-α-R	3′	TCACAGCCCAGAGAGCAGA	
5	IFN-β	NM_001003923.1	qRT-PCR-IFN-β-F	5′	TCGCTCTCCTGATGTGTTTCTC	82
			qRT-PCR-IFN-β-R	3′	AAATTGCTGCTCCTTTGTTGGT	
6	GAPDH	NM_001206359.1	qRT-PCR-GAPDH-F	5′	TGGAGTCCACTGGTGTCTTCAC	121
			qRT-PCR-GAPDH-R	3′	TTCACGCCCATCACAAACA	

### Free Fatty Acids Assay

According to the manufacturer's protocol of free fatty acids assay kit (Nanjing Jiancheng Bioengineering Institute, A042-2-1), centrifuge cell culture supernates samples for 20 min at 1,000 g and remove particulates. And then, add 50 μl of Standard or Sample to the appropriate wells. Blank well doesn't add anything. Next, add 100 μl of Enzymeconjugate to standard wells and sample wells except the blank well, cover with an adhesive strip and incubate for 60 min at 37°C. Wash the Microtiter Plate 4 times. Then, add Substrate A 50 μl and Substrate B 50 μl to each well. Gently mix and incubate for 15 min at 37°C. Protect from light. Add 50 μl Stop Solution to each well. Read the Optical Density (OD) at 450 nm using a microtiter plate reader within 15 min. Finally, the measured OD value is substituted into the curve drawn by the standard to represent the FFAs concentration of each hole. Statistical analysis and comparison were made between the two groups, *P* < 0.05 was the statistical difference.

### ATP Assays

PK-15 or 3D4/2 cells were infected with CSFV or mock infected and treated with etomoxir, TMZ, or DMSO control. According to the manufacturer's protocol of enhanced ATP assay kit (Beyotim, S0027), cells were washed twice in PBS and then incubated in ATP lysis buffer for 20 min. After pyrolysis, the supernatant was centrifuged at 12,000 rpm for 20 min at 4°C for subsequent determination. Next, ATP standard solution with appropriate concentration gradient and ATP detection solution was prepared. Then, the RLU value was determined by chemiluminometer after mixing 20 μL of test sample or ATP standard in the test tube containing 100 μL of ATP detection working fluid with a gun (micro-pipette) at least 2 s apart. Finally, the measured RLU value was substituted into the curve drawn by the standard solution to represent the ATP concentration of each hole. Statistical analysis and comparison were made between the two groups, with *P* < 0.05 as the statistical difference.

### Western Blot Analysis

Different cell samples were washed twice in cold phosphate-buffered saline (PBS) and then incubated in RIPA lysis buffer (Beyotim, P0013B) containing 1 mM PMSF (Beyotim, ST506) for 20 min. The extracted proteins were quantified by the BCA protein assay kit (Beyotim, P0012) and boiled for 10 min in 5 × SDS-PAGE sample loading buffer (Beyotim, P0015L). Equal amounts of protein samples were separated on 10% SDS-PAGE and transferred onto a polyvinylidene difluoride (PVDF) membrane. The PVDF membranes were first blocked in PBS containing 2% non-fat milk powder and 0.05% Tween 20 at 37°C for 1 h, which were then incubated with primary antibodies at 4°C overnight and then with the corresponding secondary antibodies conjugated to HRP at 37°C for 2 h. The immunolabeled protein complexes were visualized using ECL Plus kit (Beyotim, P0018), using the CanoScan LiDE 100 scanner system (Canon).

### Statistical Analysis

The data are expressed as the mean ± standard deviation (SD) and were analyzed by two-way ANOVA using the GraphPad Prism 6 software. Value of P lesser than 0.05 was considered statistically significant.

## Data Availability Statement

All datasets generated for this study are included in the article/Supplementary Material.

## Ethics Statement

The authors declare that the animal breeding, care and all experiments were conducted following regulations of the Laboratory Animal Center of South China Agricultural University and approved by the Laboratory Animal Ethics Committee of South China Agricultural University. The waste generated during the whole experimental period, including CSFV-infected cells and piglets, biochemical reagent waste liquid and other toxic substances, is uniformly recycled and harmlessly processed by South China Agricultural University.

## Author Contributions

SM designed the experiment and drafted the manuscript. QM and WC performed experiments and analyzed lipidomics data. MeZ, KW, DS, and XL carried out animal experiments. EZ, SF, LY, and HD analyzed experimental results and data. MiZ and JC guided the design of the study and revised the manuscript. All authors read and approved the final manuscript.

### Conflict of Interest

The authors declare that the research was conducted in the absence of any commercial or financial relationships that could be construed as a potential conflict of interest.
